# 2,3-*O*-Isopropylidene-*α*-d-lyxofuranose, the Monoacetone-d-lyxose of Levene and Tipson^1^

**DOI:** 10.6028/jres.065A.054

**Published:** 1961-12-01

**Authors:** Robert Schaffer

## Abstract

A proof of structure is presented for the title compound, previously uncharacterized. In the course of the investigation, the crystalline 2,3-*O*-isopropylidene acetals of calcium d-lvxonate, d-lyxono-1,4-lactone, d-lyxitol, *β*-d-erythrofuranose, calcium d-erythronate, and d-erythrono-1,4-lactone were prepared. The diacetate and dibenzoate of 2,3-*O*-isopropylidene-d-lyxose were also synthesized.

## 1. Introduction

Among the acetals whose infrared absorption spectra were studied by Tipson, Isbell, and Stewart [1]^2^ was a crystalline mono-*O*-isopropylidene-d-lyxose (I) that Levene and Tipson [2] had prepared in 1936, but whose structure has not hitherto been determined. Interestingly, its infrared absorption spectrum was found [1] to contain bands that had previously been tentatively correlated with furanoid structures [3], but, as was pointed out [1], a furanoid ring could not be assigned from this evidence alone, because some nonfuranoid structures show many of these bands, too. In addition, however, since d-lyxose is in the same homomorphous series as d-mannose, which forms a furanose 2,3:5,6-di-*O*-isopropylidene acetal [4], the mono-*O*-isopropylidene-d-lyxose could be expected to possess, structural similarities to the di-*O*-isopropylidene-d-mannose. This consideration extended the possibility that compound I would prove to be a *reducing*, *furanoid*, sugar derivative. The scarcity of compounds having such structure, and our interest in the properties that it would exhibit, prompted this study of Levene and Tipson’s compound.

## 2. Discussion

From its behavior with Fehling solution, it appeared that the lyxose derivative I is indeed a reducing sugar derivative, but the rate of reaction was so low that additional evidence was sought in order to make sure that this was simply a reaction indicative of an unsubstituted reducing group in the molecule. This was achieved by obtaining a quantitative oxidation of I with hypoiodite [5], and isolating a crystalline calcium *O*-isopropylidene-d-lyxonate dihydrate (II). Treatment of II with a cation-exchange resin 3 led to removal of the metal ıon, and this was followed by crystallization of an *O*-isopropylidene-d-lyxonic lactone (III); the latter was also obtained directly from I by oxidation with bromine in a buffered solution [6]. Additional evidence for the presence of a free reducing group was found on treating I with sodium borohydride [7]; a crystalline *O*-isopropylidene-d-lyxitol (IV) was isolated. Thus, I exhibits the properties of an aldopentose that has been condensed with an equimolecular proportion of acetone at two of its four *alcoholic* hydroxyl groups.

The rather slow reaction that took place on treating I with sodium metaperiodate provided evidence for one of the points of attachment of the isopropylidene group. No acid was detected among the products after 40 hr of reaction (when 0.7 mole of periodate had been consumed per mole of I); this indicates that the hydroxyl group at C–2 is substituted, since otherwise formic acid would have been produced [8].

The location of the second point of attachment of the isopropylidene group was determined upon characterizing compound V, a crystalline product that resulted from interaction of IV and sodium metaperiodate. This compound (V) had the composition of an acetonated tetrose. Acid hydrolysis converted it to a sirupy compound, which was found by paper chromatography to behave identically with an authentic specimen of d-erythrose [9]. Compound V was further characterized by oxidation with hypoiodite to a crystalline calcium *O*-isopropylidenetet-ronate hemihydrate (VI); and from this salt (VI), a crystalline *O*-isopropylidenetetronic lactone (VII) was prepared. Bromine in buffered solution yielded the same lactone (VII) directly from V. Acid hydrolysis converted VII to d-erythronic lactone [10], which was identified by paper chromatography. Thus, V is an acetonated d-erythrose, and, from this conclusion, the location of the isopropylidene group of compounds I through VII can be deduced. As the isopropylidene-d-lyxitol (IV) can be degraded to a d-*erythro*-tetrose by glycol cleavage only at C–4 and C–5, and as the hydroxyl group at C–1 of compound IV is unsubstituted (from its origin), therefore the isopropylidene linkages are to the oxygen atoms at C–2 and C–3. These two carbon atoms become C–3

**Figure:**
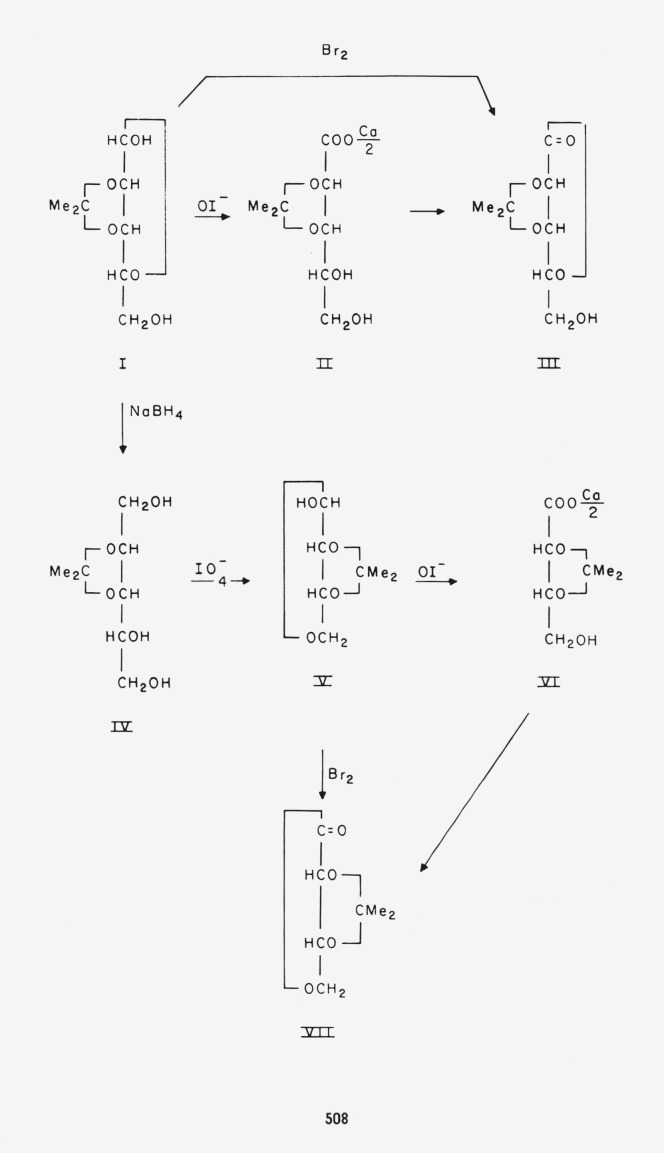

and C–2, respectively, of the derived tetrose, but, due to the symmetry of the substituent, compounds I through VII are all 2,3-*O*-isopropylidene derivatives. Of these, compounds IV and V had previously been prepared but not crystallized [11], and the enantiomorph of V was recently crystallized by Baxter and Perlin [12].

Molecular models of 2,3-*O*-isopropylidene-d-lyxose show that the aldehyde group of the acyclic sugar can form either a furanose ring with the hydroxyl group at C–4, or a pyranose ring with the hydroxyl group at C–5, and that, for both rings, *α* and *β* anomers are possible [2]. Similarly, a model of 2,3-*O*-isopropylidene-d-erythrose shows that a furanose ring, which is the only possible sugar-ring form of this acetal, can readily be constructed, and that both an *α* or a *β* anomer are possible.

The actual hemiacetal-ring structures of the acetonated sugars were ascertained from the sizes of the lactone rings of the products obtained on oxidation under conditions that precluded expansion or contraction of the lactone rings produced. These conditions were provided by a buffer, in the oxidation medium, that served to neutralize any acid that might be formed by hydrolysis of lactone and might otherwise be transformed into a lactone of another ring size. This is the method of Isbell and Hudson, and of Isbell [6], who showed that, in the presence of buffer, a cyclic sugar is oxidized directly to a lactone of the same ring size. In the present study, evidence was educed to show that the bromine-oxidation product of each sugar is the lactone that was subsequently isolated. The ring structures of the isolated lactones, which are new compounds, were deduced [13] from their carbonyl stretching frequencies in the infrared. The carbonyl stretching frequencies given in the literature are in the range of 1,725 to 1,760 cm^−1^ for *δ*-lactones (and for esters) and in the range of 1,755 to 1,800 cm^−1^ for *γ*-lactones. Compound III, with an absorption maximum at 1,785 cm^−1^, and compound VII, with a maximum at 1,770 cm^−1^, fall in the latter range and are, therefore, *γ*-lactones. (The closely related 2,3:5,6-di-*O*-isopropylidene-d-mannono-1, 4-lactone absorbs at 1,780 cm^−1^ [11].)

In determining the hemiacetal-ring form of compound I, a solution of the products obtained on reaction of I with bromine at 0° for 2.5 hr, when the oxidation was about complete, was found to have a specific optical rotation of +100°. A solution of the isolated oxidation product, compound III, had an initial specific rotation of +106°, and on allowing both of the above solutions to stand, their values decreased. In contrast, the free acid form of III and its calcium salt have values of −22 and −46.5°, respectively, and, on standing in solution, the rotation of the former becomes dextrorotatory. Thus, the optical rotatory properties of the oxidation mixture correspond closely to those of III, which was obtained in high yield simply by extracting the buffered oxidation mixture; and III is, therefore, the directly formed oxidation product. Compound III is 2,3-*O*-isopropylidene-d-lyxono-1,4-lactone and, consequently, compound I is a 2,3-*O*-isopropylidene-d-lyxofuranose. Compound I shows an initial ${\left[\alpha \right]}_{D}^{22}$ of +21.4° that changes in 10 min to +18.5°. From this mutarotation, an *α* configuration is assigned [14] to C–1 of I.

Starting with compound V, the specific optica rotation of the products, measured after about 2 hr of oxidation by bromine, was −103°; for a freshly prepared solution of VII, the isolated product, the value is −116°. With time, the rotations of both of these solutions become less levorotatory. On the other hand, the free acid form of VII and its calcium salt have specific optical rotations of +22.2 and + 65.6°, respectively, and the rotation of the acid becomes levorotatory on standing in solution. Compound VII, which is isolated in very high yield by extraction of the reaction products, is clearly the direct product of oxidation of V. Compound VII is 2,3-*O*-isopropylidene-d-erythrono-1,4-lactone, and V, its precursor, is, therefore, a 2,3-*O*-isopropylidene-d-erythrofuranose. Although mutarotation was not detected for its enantiomorph [10], compound V *was found to mutarotate* from ${\left[\alpha \right]}_{D}^{22}-68$ to −66° in water and from ${\left[\alpha \right]}_{D}^{24}-77.6$ to −76.2° in methanol. Therefore, it is 2,3-*O*-isopropylidene-*β*-d-erythrofuranose [14].

A partial explanation of the relatively slow oxidation of I by periodate can be advanced, now that its structure is known. For the reaction to proceed, hydroxyl groups at C–4 and C–5 are needed, and both are present on tautomerization of the hemiacetal form to the open-chain form of I. These vicinal hydroxyl groups should be as reactive as the corresponding pair in compound IV, whose reaction with periodate was complete in less than 5 min. The much lower rate actually observed with I must, because of the observed mutarotation, be due to an exceedingly low equilibrium concentration of the open-chain form. This explanation for the diminished rate of periodate oxidation of I, because of the presence of a furanose ring, is in contrast, but not in conflict, with the acceleration of the periodate reaction that may accompany formation of a furanose ring, since the latter oxidation occurs with *α,γ*-dihydroxy aldehydes (where the bond between the anomeric hydroxyl group of the furanose ring formed and the *α*-hydroxyl group is oxidized [8]).

Two crystalline esters of 2,3-*O*-isopropylidene-d-lyxose were synthesized, namely, a diacetate and a dibenzoate. As they were obtained in high yield on treating pyridine solutions of I with acetic anhydride and with benzoyl chloride, respectively, it is assumed that they are 1,5-diesters. From steric considerations, the anomeric acyl groups are expected to be *exo.* Thus, they are tentatively formulated as 1,5-di-*O*-acyl-2,3-*O*-isopropylidene-*α*-d-lyxofuranoses.

As pointed out by Levene and Tipson [2], and confirmed in this laboratory, on slow distillation of I, there is formed a second volatile fraction, which distills at temperatures higher than does I. This as-yet-uncrystallized material is dextrorotatory [2] and nonreducing. Its constitution will be the subject of a future report.

## 3. Experimental Procedures

### 3.1. Acetonation of d-Lyxose to 2,3-*O*-Isopropylidene-*α*-d-lyxofuranose (I)

The procedure of Levene and Tipson [2] was modified by use of a magnetic stirrer for mixing 20 g of crystalline d-lyxose, 50 g of anhydrous cupric sulfate, and 0.5 ml of concentrated sulfuric acid in 500 ml of technical acetone in a stoppered Erlenmeyer flask. The heat from the stirrer kept the mixture at about 35° for the 5-day reaction time. The mixture was then filtered, the filtrate was passed through 40 ml of acetone-washed, anionexchange resin,^4^ and the effluent was evaporated to dryness under diminished pressure. The residue was distilled at 0.04 mm, and a fraction weighing 6.0 g was collected at 105 to 127°. Compound I crystallized from its solution in toluene; yield 4.3 g; mp 81.5–82.5°; ${\left[\alpha \right]}_{D}^{22}+21.4{}^{\circ}$ (extrapolated) →+18.5° in 10 min (*c*, 10; water). Compound I had been reported with bp 110° at 0.1 mm, mp 79 to 80°, and ${\left[\alpha \right]}_{D}^{22}+18.7{}^{\circ}$ at equilibrium in water [2]. A second fraction, weighing 7.4 g, distilled between 170 and 180°; this higher-boiling fraction is a viscous sirup that does not reduce Fehling solution unless it is first hydrolyzed with dilute mineral acid.

Compound I, heated with Fehling solution, does not begin to show visible evidence of reaction until many minutes have elapsed. Cuprous oxide is then slowly precipitated, and its amount gradually increases over several hours.

A sample (0.020 g) of I, treated with 0.053 g of sodium metaperiodate in 10 ml of solution at room temperature, showed 0.1, 0.5, and 0.7 mole of periodate consumed per mole of I, in 2, 17, and 40 hr of reaction, respectively. On adding a droplet of ethylene glycol to a 1-ml aliquot of the mixture after 40 hr of reaction, one drop of 0.01 *N* sodium hydroxide brought the solution to the endpoint of bromothymol blue.

### 3.2. Calcium 2,3-*O*-Isopropylidene-d-lyxonate Dihydrate (II)

An aqueous solution of 0.95 g of I was treated alternately with 10 ml of 0.1 *N* iodine solution (0.25 *N* potassium iodide and 0.1 *N* iodine) and (dropwise) with 15 ml of 0.1 *N* sodium hydroxide, until a total of 110 ml of the former and 165 ml of the latter had been added. The additions required about 15 min, and, after 5 min additional, the mixture was cooled to 0° and passed through a column containing 60 ml of ice-cold, cation-exchange resin (see footnote 3). The effluent was collected in a stirred, aqueous slurry of 10 g of silver carbonate. After filtration of the suspension, the ice-cold filtrate was passed through an additional 10 ml of the resin. Neutralization of the effluent with calcium hydroxide was followed by concentration under diminished pressure and crystallization of II from 95-percent ethyl alcohol. The product was purified further by dissolution in absolute ethanol, filtration, evaporation, and recrystallization from 95-percent ethyl alcohol. Calcium 2,3-*O*-isopropylidene-d-lyxonate dihydrate was isolated in 80-percent yield; ${\left[\alpha \right]}_{D}^{22}-46.5{}^{\circ}$ (*c*, 1; water).

#### Anal

Calculated for C_16_H_26_CaO_12_·2 H_2_O: C, 39.5; H, 6.2; Ca, 8.2. Found: C, 39.2; H, 6.3; Ca, 8.2.

Acidification of a solution of 0.122 g of the salt with an equivalent proportion of hydrochloric acid changed the specific rotation to ${\left[\alpha \right]}_{D}^{22}-22{}^{\circ}$ (calculated as 2,3-*O*-isopropylidene-d-lyxonic acid), and no change in optical rotation was detected during the next 5 min. However, on addition of a second equivalent proportion of acid, the observed optical rotation^5^ changed in several hours from −1.24 °S to values higher than +2.9 °S, then decreased slowly, and finally increased very slowly during the next 10 days to +2.6 °S.

### 3.3. 2,3-*O*-Isopropylidene-d-lyxono-1,4-lactone (III) From II

An ice-cold aqueous solution of 0.68 g of II was passed through a column containing 10 ml of ice-cold, cation-exchange resin (see footnote 3), and the effluent was freeze-dried. The dried residue was crystallized from benzene; yield, 90 percent; mp 99 to 100°; ${\left[\alpha \right]}_{D}^{22}+106{}^{\circ}$, initially (*c*, 1.6; water). The initial observed rotation, +9.9 °S, decreased to +3.6 °S in 22 days.

#### Anal

Calculated for C_8_H_12_O_5_: C, 51.0; H, 6.4. Found C, 51.1; H, 6.4.

### 3.4. Preparation of Compound III by Bromine Oxidation of I

A sample (0.19 g) of I, mixed with 1.2 g of barium carbonate, was treated at 0° with 20 ml of an ice-cold solution containing 1.2 g of barium bromide dihydrate, 0.4 ml of bromine, and 0.08 ml of concentrated hydrobromic acid. After 2.5 hr, ethylene was bubbled through the mixture, and the debrominated suspension was filtered. The specific optical rotation of the filtrate was about +100° and changed to +96° in the next 25 min. The filtrate was freeze-dried, and the residue, extracted with chloroform, gave, on evaporation of the extract, 0.16 g of crystalline III.

### 3.5. 2,3-*O*-Isopropylidene-d-lyxitol (IV)

To a stirred solution of 1.0 g of I in 5 ml of water at 0°, 0.175 g of sodium borohydride was added, and the solution was allowed to warm to room temperature. The next day, the solution was again cooled to 0°, treated with 5 ml of ice-cold, cation-exchange resin (see footnote 3), and then passed through a column containing an additional 5 ml of the ice-cold resin. The effluent was concentrated under diminished pressure (with absolute ethanol, to remove water), and the concentration was repeated several times with methanol added (to remove boric acid as methyl borate). Crystalline IV was obtained in 80-percent yield from a solution in ethanol by the addition of pentane; mp 84.5–85.5°; ${\left[\alpha \right]}_{D}^{22}+10{}^{\circ}$ (*c*, 2; water). A sirupy IV was reported by Ballou [11].

#### Anal

Calculated for C_8_H_16_O_5_: C, 50.0; H, 8.4. Found: C, 50.2; H, 8.6.

### 3.6. 2,3-*O*-Isopropylidene-*β*-d-erythrofuranose (V)

A solution of 0.192 g of IV, treated with 0.225 g of sodium metaperiodate, showed within 5 min ${\left[\alpha \right]}_{D}^{22}-66{}^{\circ}$ (calculated as V), and the rotation did not change in the next hour. For isolation of crystalline V, 0.95 g of I in 20 ml of water was reduced at room temperature with 0.1 g of sodium borohydride. After the solution had been kept overnight, carbon dioxide was bubbled through it for 1 hr, and then 1.18 g of sodium metaperiodate was added. One hour later, the mixture was repeatedly extracted with ether. The combined extracts were evaporated to dryness, to yield a crystalline residue. This was redissolved in ether, and the solution was filtered and re-evaporated. For purification, the product was sublimed at 0.03 mm at a bath temperature of 52°. An ether solution of the product gave, on drying, crystalline V; weight 0.7 g; mp 32.5–34.5°; ${\left[\alpha \right]}_{D}^{22}-68{}^{\circ}\to -66{}^{\circ}$ in 2 min (*c*, 4.2; water); ${\left[\alpha \right]}_{D}^{22}-77.6{}^{\circ}\to -76.2{}^{\circ}$ in 3 hr (*c*, 7; methanol). Sirupy V has been reported [11], and mp 29 to 31° and ${\left[\alpha \right]}_{D}^{20}+72{}^{\circ}$ (in methanol) were reported [12] for the crystalline enantiomorph of V.

#### Anal

Calculated for C_7_H_12_O_4_: C, 52.4; H, 7.5. Found: C, 52.6; H, 7.7.

A sample of V in water containing cation-exchange resin (see footnote 3) was heated at 80° for 1 hr. The hydrolyzate was chromatographed on paper. The compound migrated like an authentic specimen of d-erythrose that had been prepared from crystalline, “dimeric” 2,4-*O*-ethylidene-d-erythrose [9].

### 3.7. Calcium 2,3-*O*-Isopropylidene-d-erythronate Hemihydrate (VI)

The oxidation of 0.16 g of V with a mixture of 22 ml of 0.1 *N* iodine solution and 35 ml of 0.1 N sodium hydroxide was carried out as described in section 3.2. The crystalline product was obtained from 95-percent ethyl alcohol; weight, 0.17 g; ${\left[\alpha \right]}_{D}^{22}+65.6{}^{\circ}$ (*c*, 1; water). It is hygroscopic; a sample was dried at 110° under vacuum before analysis.

#### Anal

Calculated for C_14_H_22_CaO_10_·0.5 H_2_O: C, 42.1; H, 5.8; Ca, 10.0. Found: C, 42.3; H, 5.8; Ca, 10.2.

A solution (9 ml) of 0.0975 g of VI in water, on treatment with 1 ml of *N* hydrochloric acid, showed ${\left[\alpha \right]}_{D}^{22}+22.2{}^{\circ}$ (1=2 dm) initially (calculated for2,3-*O*-isopropylidene-d-erythronic acid). After 4 hr, the observed rotation (see footnote 5) was −1.7 °S, and, during the next 16 hr, the rotation at first continued to become more levorotatory and then less levorotatory; a final rotation of −1.25 °S was observed.

### 3.8. 2,3-*O*-Isopropylidene-d-erythrono-1,4-lactone (VII) From VI

A solution of 0.1 g of VI in ice-cold water was passed through a column of 5 ml of ice-cold, ionexchange resin (see footnote 3), and was then concentrated under diminished pressure to a thin sirup and reconcentrated several times with absolute ethanol; VII crystallized from the sirup. The product was recrystallized from ice-cold ether; yield 0.06 g; mp 65 to 67.5°; initial ${\left[\alpha \right]}_{D}^{22}-116{}^{\circ}$ (*c*, 1; water). The rotation (see footnote 5), observed in a 2-dm tube, changed to −1.2 °S in 6 days.

#### Anal

Calculated for C_7_H_10_O_4_: C, 53.2; H, 6.4. Found: C, 53.0; H, 6.4.

A sample of VII in water was hydrolyzed by means of a cation-exchange resin (see footnote 3) at 80° for 2 hr, and paper chromatography of the hydrolyzate showed a product that migrated like d-erythrono-1,4-lactone [10].

### 3.9. Preparation of VII by Bromine Oxidation of V

A stirred solution of 0.164 g of V in 10 ml of ice-cold water was treated with 1.2 g of barium carbonate, and with 10 ml of a solution containing 1.2 g of barium bromide dihydrate, 0.4 ml of bromine, and 0.08 ml of concentrated hydrobromic acid. After 2 hr, the mixture was debrominated with ethylene, and filtered; the filtrate had ${\left[\alpha \right]}_{D}^{22}-103{}^{\circ}$, and this slowly changed to less levorotatory values. The solution was quickly extracted several times with chloroform, and evaporation of the combined extracts gave VII in about 90-percent yield.

### 3.10. 1,5-Di-*O*-acetyl-2,3-*O*-isopropylidene-*α*-d-lyxofuranose

A solution of 1 ml of acetic anhydride in 9 ml of pyridine rapidly dissolved 0.3 g of I. After 18 hr at room temperature, 50 g of ice (from distilled water) was added, and the mixture was stirred until the ice had melted; the solution was then freeze-dried. On dissolving the sirupy residue in 1.0 ml of absolute ethanol, cooling the solution to 0°, and adding water dropwise, the crystalline diacetate was obtained in 90-percent yield; mp 49.5–50.5°; ${\left[\alpha \right]}_{D}^{22}+62.8{}^{\circ}$ (*c*, 1; chloroform).

#### Anal

Calculated for C_12_H_18_O_7_: C, 52.5; H, 6.6. Found: C, 52.3; H, 6.5.

### 3.11. 1,5-Di-*O*-benzoyl-2,3-*O*-isopropylidene-*α*-d-lyxofuranose

A solution of 0.25 g of I in 8 ml of pyridine and 2 ml of benzoyl chloride was kept at 0° for several hours and then at room temperature for 3 days. The mixture was poured into 100 ml of ice and water, and, after being stirred for 2 hr, was extracted with chloroform. The combined extracts were successively washed with aqueous sulfuric acid, aqueous sodium bicarbonate solution, and water, and then dried. The product was crystallized from ethanol; yield 0.3 g; mp 93.5 – 94.5°; ${\left[\alpha \right]}_{25}^{D}+17.3{}^{\circ}$ (*e*, 1; chloroform).

#### Anal

Calculated for C_22_H_22_O_7_: C, 66.3; H, 5.6. Found: C, 66.1; H, 5.6.

## Footnotes

2 Figures in brackets indicate the literature references at the end of this paper.

3 Amberlite IR–120 (H^+^), a product of Rohm and Haas Co., Philadelphia, Pa.

4 Duolite A-4, a product of Chemical Process Co., Redwood City, Calif.

5 Observed optica] rotations, measured in a saccharimeter, are given in “degrees sugar” (°S). One degree circular equals 0.3462 times one degree sugar.
